# Operational Determination of Subjective Cognitive Decline, Mild Cognitive Impairment, and Dementia Using Sum of Boxes of the Clinical Dementia Rating Scale

**DOI:** 10.3389/fnagi.2021.705782

**Published:** 2021-09-07

**Authors:** Yu-Wan Yang, Kai-Cheng Hsu, Cheng-Yu Wei, Ray-Chang Tzeng, Pai-Yi Chiu

**Affiliations:** ^1^Department of Neurology, China Medical University Hospital, Taichung, Taiwan; ^2^Department of Medicine, China Medical University, Taichung, Taiwan; ^3^Artificial Intelligence Center for Medical Diagnosis, China Medical University Hospital, Taichung, Taiwan; ^4^Department of Exercise and Health Promotion, College of Kinesiology and Health, Chinese Culture University, Taipei, Taiwan; ^5^Department of Neurology, Tainan Municipal Hospital, Tainan, Taiwan; ^6^Department of Neurology, Show Chwan Memorial Hospital, Changhua, Taiwan; ^7^Department of Nursing, College of Nursing and Health Sciences, Da-Yeh University, Changhua, Taiwan

**Keywords:** sum of boxes of the Clinical Dementia Rating, normal cognition, subjective cognitive decline, mild cognitive impairment, dementia

## Abstract

**Objectives:** The Clinical Dementia Rating (CDR) Scale is the gold standard for the staging of dementia due to Alzheimer's disease (AD). However, the application of CDR for the staging of subjective cognitive decline (SCD) and mild cognitive impairment (MCI) in AD remains controversial. This study aimed to use the sum of boxes of the CDR (CDR-SB) plus an SCD single questionnaire to operationally determine the different stages of cognitive impairment (CI) due to AD and non-AD.

**Methods:** This was a two-phase study, and we retrospectively analyzed the Show Chwan Dementia registry database using the data selected from 2015 to 2020. Individuals with normal cognition (NC), SCD, MCI, and mild dementia (MD) due to AD or non-AD with a CDR < 2 were included in the analysis.

**Results:** A total of 6,946 individuals were studied, including 875, 1,009, 1,585, and 3,447 with NC, SCD, MCI, and MD, respectively. The cutoff scores of CDR-SB for NC/SCD, SCD/MCI, and MCI/dementia were 0/0.5, 0.5/1.0, and 2.5/3.0, respectively. The receiver operating characteristic (ROC) analysis showed that the area under the curve (AUC) values of the test groups were 0.85, 0.90, and 0.92 for discriminating NC from SCD, SCD from MCI, and MCI from dementia, respectively. Compared with the Cognitive Abilities Screening Instrument or the Montreal Cognitive Assessment, the use of CDR-SB is less influenced by age and education.

**Conclusion:** Our study showed that the operational determination of SCD, MCI, and dementia using the CDR-SB is practical and can be applied in clinical settings and research on CI or dementia.

## Introduction

The Clinical Dementia Rating (CDR) Scale is a semi-structured interview and a golden standard protocol for investigating people with dementia of the Alzheimer type or probable Alzheimer's disease (AD) (Hughes et al., 1982; Morris, 1997). Many studies have indicated that non-medical personnel reliably apply the CDR Scale to identify and determine the stage of dementia (McCulla et al., 1989; Chaves et al., 2007). Based on the combination of clinical history acquisition and a simple cognitive test, the CDR has become widely used in longitudinal studies and clinical trials to standardize the staging of AD and related disorders (Vos et al., 2013; Williams et al., 2013).

The pre-dementia phases of AD or other dementia, which include subjective cognitive decline (SCD) and mild cognitive impairment (MCI), have gradually attracted the attention of researchers; therefore, robust studies for predicting progression among people in these phases are in full swing (Palmer et al., 2007; Mitchell and Shiri-Feshki, 2009; Snitz et al., 2018; Mazzeo et al., 2019). Although the CDR has been used in many clinical trials or studies, its reliability is suboptimal in very mild dementia (VMD) cases with CDR = 0.5 (Rockwood et al., 2000; Schafer et al., 2004; Duara et al., 2010). However, many studies have indicated that the sum of boxes of the CDR (CDR-SB) can provide additional information to the CDR in detecting VMD and distinguishing these patients from patients with MCI (Lynch et al., 2006; Julayanont and DeToledo, 2020). The CDR-SB score has a wider dynamic range (0–18) than the CDR (0–3) and can be analyzed as an interval variable.

Evidence of CDR utility in dementia diagnosis is robust (McCulla et al., 1989; Morris, 1997; Lynch et al., 2006; Chaves et al., 2007; O'Bryant et al., 2008; Vos et al., 2013; Williams et al., 2013; Julayanont and DeToledo, 2020; Huang et al., 2021). However, the clinical application of CDR-SB in cognitive evaluation among people with SCD or MCI is still lacking. Moreover, when diagnosing dementia due to non-AD, that is, dementia with Lewy bodies (DLB), vascular dementia (VaD), or dementia due to Parkinson's disease (PDD), the question of using the CDR in AD remains to be answered. Less clinical evidence has been provided, and some modifications may be necessary (Lima-Silva et al., 2020).

In our recent experience and studies using artificial intelligence (AI) for the diagnosis of cognitive impairment (CI) and dementia, the CDR and CDR-SB have become perfect references for machine learning in our newly designed questionnaires (Chiu et al., 2019a; Chang et al., 2020; Zhu et al., 2020a,b). Therefore, we highly recommend CDR as a further AI study. For future studies that compare individuals with NC, SCD, and MCI on the risks, prevention, and follow-up for progression to dementia, a reliable, simple, and useful tool is urgently needed. The CDR, which assesses the patient cognition and acquires daily function from their caregivers, is a good candidate staging tool that can fulfill the criteria for clinically urgent and unmet needs.

To test the clinical utility of the CDR-SB for the diagnosis of the different CI stages of AD and non-AD, we designed and analyzed the data from a relatively large dementia database that has been created for registering more than 10,000 individuals. Each individual had detailed clinical data, such as demographic, clinical history, CDR, cognitive and neuropsychological tests, brain imaging, and laboratory findings. Nearly all of them had a definite diagnosis of CI stages and disease subtypes identified by well-experienced neurologists or a consensus group composed of neurologists, psychiatrists, neuroradiologists, nuclear medicine physicians, neuropsychologists, and technicians. CI diagnoses were made using the CDR and other neuropsychological tests. Criteria for AD or vascular cognitive impairment (VCI) were established by the National Institute on Aging and Alzheimer's Association Workgroup (NIA-AA) (McKhann et al., 2011) or the 2011 American Heart Association (AHA) criteria. DLB, Parkinson's disease (PD), PDD, and other disorders (OD) were diagnosed according to the clinical or diagnostic criteria for those disorders. We aimed to define the operational criteria for the differential diagnosis of normal cognition (NC), SCD, MCI, and dementia by determining the cutoff scores using the CDR-SB. In addition, in this study, we hope to provide further evidence of practical diagnostic criteria studying the different CI stages by using the CDR-SB in AD and other brain disorders.

## Materials and Methods

This study is a retrospective analysis of the dementia registry database from the Show Chwan Healthcare System, currently applied in three centers in Taiwan (two in central Taiwan and one in southern Taiwan). In the database, the detailed clinical history of each participant was recorded using a structured questionnaire called the History-Based Artificial Intelligent Clinical Dementia Diagnostic System (HAICDDS), which has been well-validated (Lin et al., 2018; Chiu et al., 2019a,b, 2020; Tsai and Chiu, 2019; Chang et al., 2020; Wang et al., 2020; Zhu et al., 2020a,b; Huang et al., 2021). In addition, CDR was used for staging dementia, and the daily function was assessed using the Instrumental Activities of Daily Living (IADL) Scale (Lawton and Brody, 1969) and Barthel Index (BI) (Mahoney and Barthel, 1965). Cognitive function was assessed using the Cognitive Abilities Screening Instrument (CASI) (Teng et al., 1994) and Montreal Cognitive Assessment (MoCA) (Freitas et al., 2012). Neuropsychiatric symptoms were assessed using the Neuropsychiatric Inventory (NPI) (Cummings et al., 1994). The CASI and MoCA for all participants were performed by trained neuropsychologists. Informants of the participants were interviewed by well-trained neuropsychologists to perform CDR, IADL, BI, and NPI. Diagnosis of the severity and subtype of dementia or CI was performed by neurologists after the clinical data, such as clinical history, neuropsychological tests, brain imaging, and laboratory data, were completed. Difficult or undetermined cases were discussed in the consensus meetings.

### Diagnosis of NC, SCD, MCI, and Dementia

Normal cognition was diagnosed when the participant had a global CDR (Morris, 1993) score of 0 and without subjective memory or other cognitive complaints. Cognitive tests, such as MoCA and CASI, should be in the normal range. The cutoff scores of the NC/MCI of the CASI (83/82) and the MoCA (20/19) were derived from our previous study published in 2019 (Chiu et al., 2019b). SCD was diagnosed when the participant had a global CDR score of 0 or 0.5 and subjective memory or other cognitive complaints. The cognitive tests, such as MoCA and CASI, should also be in the normal range as in NC. MCI was diagnosed based on the criteria for MCI of the NIA-AA in 2011 (Albert et al., 2011) as a change in cognition with impairment in one or more cognitive tests (CASI or MoCA), but there is no evidence of impairment in social or occupational functioning (IADL > 6) with a CDR score of 0.5. The cutoff scores of MCI/dementia of the CASI (74/73) and the MoCA (14/13) were also derived from the same study published in 2019 (Chiu et al., 2019b). Dementia diagnosis was made according to the criteria for dementia developed by the NIA-AA (Kim et al., 2017). Participants with dementia had impairments in two or more cognitive domains and a decline in daily function (IADL < 7).

### Diagnosis of Disease Subtypes

Alzheimer's disease was diagnosed according to the criteria for probable AD developed by the NIA-AA (McKhann et al., 2011). Participants who had evidence of insidious onset and progressive cognitive decline on subsequent evaluations based on information from informants and cognitive testing in the amnestic or other cognitive domains were diagnosed as patients with AD. Vascular cognitive disorder (VCD)/VaD diagnosis was determined according to the 2011 AHA criteria for VCD (Gorelick et al., 2011). CI and clear imaging evidence for vascular disease were available. CI should be due to vascular disease with a temporal relationship between vascular disease and the onset of cognitive deficits. DLB diagnosis was made according to the revised consensus criteria for probable DLB developed by the fourth report of the DLB consortium (McKeith et al., 2017). For probable DLB diagnosis, “two or more core clinical features” or “one core clinical feature, but with one or more indicative biomarkers” is required. Core clinical features include fluctuation in cognition, visual hallucinations, spontaneous parkinsonism, and rapid eye movement (REM) sleep behavior disorder. Indicative biomarkers include abnormal uptake in the metaiodobenzylguanidine scintigraphy and dopamine transporter imaging and REM sleep without atonia in polysomnography. PD/PDD diagnosis was processed according to the 2015 (Postuma et al., 2015) and 2007 (Emre et al., 2007) criteria for probable PD and PDD, respectively. Participants should have parkinsonism, defined as bradykinesia, in combination with either resting tremor, rigidity, or both. Clinically probable PD requires the absence of absolute exclusion criteria and the presence of red flags counterbalanced by supportive criteria. CI or dementia should be diagnosed after a well-established PD for more than 1 year. OD diagnosis with CI or dementia was determined according to their clinical or diagnostic criteria.

### Study Procedures

This study is a two-phase study that included the design and test phases. In the design phase, the data from 2015 to 2017 were analyzed. Individuals with NC, SCD, MCI, and dementia due to AD and those without AD were included in the analysis. Non-AD groups included cerebrovascular disease (CVD), VCI, PD with and without dementia, DLB, and OD. The cutoff scores of the CDR-SB for NC/SCD/MCI/mild dementia (MD) were derived.

In the test phase, the data from 2018 to 2020 were analyzed. This study aimed to separate NC/SCD/MCI due to vascular disease, PD, or other brain disorders from pure NC/SCD/MCI without brain disorders. According to clinical criteria or studies, individuals with established brain disorders are essential in diagnosing the progression to the same disease entity (Vanneste, 2000; Starkstein and Jorge, 2005; Emre et al., 2007; Gorelick et al., 2011). However, most of the pure NC/SCD/MCI without other brain disorders are considered to progress to AD instead of VaD, Lewy body disease (LBD), or dementia due to the existing brain disorder (Hsiung et al., 2006; Maioli et al., 2007; Rountree et al., 2007). Therefore, pure NC/SCD/MCI and NC/SCD with vascular disease and vascular CI/no dementia (VCIND) were divided into the AD and VCI/VaD groups, respectively. PD with NC/SCD/MCI or DLB and NC/SCD/MCI with other well-diagnosed brain disorders were divided into the LBD and OD groups, respectively. The NC/SCD/MCI diagnostic criteria in all disease groups were based on the neurocognitive tests with the cutoff scores described in the “Diagnosis of NC, SCD, MCI, and dementia” section. Individuals with NC, SCD, MCI, and MD due to AD or non-AD with a global CDR < 2 were included in the analysis. The cutoff scores of the CDR-SB for NC/SCD/MCI/MD derived from the design phase were all tested in the AD and non-AD groups.

### Statistics

The Chinese version of SPSS 22.0 for Windows (IBM, SPSS Inc., Chicago, IL, USA) was used for the statistical analyses. The demographic data in the design phase are summarized descriptively. Comparisons of the demographic data, neuropsychological tests, CDR-SB, IADL, CASI, BI, MoCA, and a composite score of the NPI (Cummings, 1988) were compared between the different groups and analyzed using one-way ANOVA with either Bonferroni or Dunnett's T3 *post-hoc* analysis according to the homogeneity of variance. Sex, CDR, and CI stages and subtypes were analyzed using the chi-square test. Youden's index was applied (maximum = sensitivity ± specificity −1) to determine the cutoff scores for differentiating between the cognitive stages. Spearman's correlation coefficients among age, education, CDR-SB, and CI stages using CDR-SB, MoCA, CASI, and NPI were summarized.

## Results

### Design Phase

The demographic data of participants in the design phase are summarized in Table 1.

**Table 1:** Demographic data of the participants in the design phase.

	**Mean (SD)**
*N*	3,498
Age, years	74.5 (11.4)
Sex, female, *N* (%)	1,975 (56.3)
Education, years	5.1 (4.7)
CDR, 0/0.5/1/2/3, *N*	294/1390/881/624/309
CDR-SB	5.9 (5.3)
CASI	54.4 (28.2)
MoCA	11.2 (8.5)
IADL	4.2 (3.3)
BI	76.6 (34.4)
NPI	8.2 (10.7)
CI severity, NC/SCD/MCI/Dementia	233/268/599/2398
Subtypes, AD/VCI/LBD/OD	1622^*^/777/793/306
Cutoff scores, NC/SCD/MCI/Dementia	
AD group	0/0.5/1.0/2.5
Non-AD group	0/0.5/1.0/3.0
All participants	0/0.5/1.0/2.5

*N, number; SD, Standard deviation; CDR, Clinical Dementia Rating Scale; CDR-SB, Sum of boxes of the Clinical Dementia Rating Scale; CASI, Cognitive Abilities Screening Instrument; MoCA, Montreal Cognitive Assessment; IADL, Instrumental Activities of Daily Living; BI, Barthel Index; NPI, Neuropsychiatric Inventory; CI, cognitive impairment; NC, normal cognition; SCD, subjective cognitive decline; MCI, mild cognitive impairment; AD, Alzheimer's disease; VCI, vascular cognitive impairment; LBD, Lewy body disease; OD, other disorders*.

* *Participants with NC/SCD/MCI without the cerebrovascular disease (CVD), LBD, or other brain disorders were divided and analyzed in the AD group*.

A total of 3,498 individuals were included in the design phase. Among them, 233 had NC, 268 had SCD, 599 had MCI, and 2,398 had dementia. According to the diagnosed disease subtypes, 1,622 had AD or NC/SCD/MCI that were not due to VCI, PD, or OD, 777 with CVD/VCI, 793 with LBD (PD/DLB), and 306 with OD, which included 126 with NPH, 106 with TBI, 27 with frontotemporal dementia (FTD), 11 with primary progressive aphasia, and 36 with other diseases. The comparison of demographic characteristics showed significant differences at different stages of CI (Table 1).

The cutoff scores of CDR-SB for NC/SCD, SCD/MCI, and MCI/MD in the AD group were 0/0.5, 0.5, 1.0, and 2.0/2.5, respectively. The receiver operating characteristic (ROC) analysis showed that the area under the curve (AUC) in this group was 0.87, 0.86, and 0.95 for discriminating SCD from NC, MCI from SCD, and dementia from MCI, respectively.

The cutoff scores of CDR-SB for NC/SCD, SCD/MCI, and MCI/MD in the non-AD group were 0/0.5, 0.5, 1.0, and 2.5/3.0, respectively. The ROC analysis showed that the AUC in this group was 0.92, 0.97, and 0.96 for discriminating SCD from NC, MCI from SCD, and dementia from MCI, respectively.

The cutoff scores of CDR-SB for NC/SCD, SCD/MCI, and MCI/MD in all the participants were 0/0.5, 0.5, 1.0, and 2.0/2.5, respectively. The ROC analysis showed that the AUC in this group was 0.89, 0.90, and 0.96 for discriminating SCD from NC, MCI from SCD, and dementia from MCI, respectively.

### Test Phase

The demographic data of participants in the test phase are summarized in Table 2.

**Table 2:** Comparison of the demographic data among participants with normal cognition, subjective cognitive decline, mild cognitive impairment, and mild dementia in the test phase.

	**NC, mean (SD)**	**SCD, mean (SD)**	**MCI, mean (SD)**	**MD, mean (SD)**	**f/χ^2^**	***p*-value**
N	642	741	986	2,012		
Age, years	63.2 (13.6)	66.3 (12.0)	70.4 (11.0)	77.2 (10.1)	345.3	<0.001^a^
CDR, 0/0.5/1	642/0/0	248/493/0	0/986/0	0/1045/967	4516.2	<0.001^a^
CDR-SB	0.0 (0.1)	0.4 (0.3)	1.3 (0.6)	4.4 (2.4)	1990.5	<0.001^b^
Female, *N* (%)	292 (45.5)	381 (51.4)	478 (47.5)	1170 (58.2)	44.8	<0.001^c^
Education	9.9 (6.9)	8.9 (4.6)	7.5 (5.4)	4.7 (4.8)	242.3	<0.001^d^
IADL	7.8 (0.9)	7.8 (0.6)	7.0 (1.5)	3.2 (2.6)	1830.9	<0.001^e^
MoCA	23.0 (5.9)	22.3 (5.3)	18.3 (5.3)	9.0 (5.4)	1864.0	<0.001^e^
CASI	85.7 (13.2)	85.4 (9.3)	78.2 (9.6)	52.0 (18.3)	1622.5	<0.001^e^
NPI	2.1 (4.0)	2.9 (4.6)	4.9 (7.2)	6.7 (8.9)	90.2	<0.001^b^
Diagnostic group AD/VCI/LBD/OD	436^*^/77/107/22	544^*^/73/104/20	650^*^/102/177/57	1036/412/390/174	179.6	<0.001^f^

*NC, normal cognition; SCD, subjective cognitive decline; MCI, mild cognitive impairment; MD, mild dementia; AD, Alzheimer's disease; VCI, vascular cognitive impairment; LBD, Lewy body disease; OD, other disorders; CDR, Clinical Dementia Rating Scale; CDR-SB, Sum of boxes of the Clinical Dementia Rating Scale; IADL, Instrumental Activities of Daily Living; MoCA, Montreal Cognitive Assessment; CASI, Cognitive Abilities Screening Instrument; NPI, Neuropsychiatric Inventory*.

* *Participants with NC/SCD/MCI without CVD, parkinsonism, or other brain disorders were divided and analyzed in the AD group. Post-hoc analysis: a: NC < SCD < MCI < MD; b: NC = SCD < MCI < MD; c: NC < SCD < MD, NC = MCI, SCD = MCI, MCI < MD; d: NC > SCD > MCI > MD; e: NC = SCD > MCI > MD; f: NC = SCD ≠ MCI ≠ MD*.

A total of 4,381 individuals were included in the test phase. For the diagnosis of CI stages in all participants, 642 patients with NC, 741 with SCD, 986 with MCI, and 2,012 with MD were included. For the diagnosis of normal or CI without other diseases, 436 patients had NC, 544 had SCD, and 650 had MCI. For the diagnosis of normal or CI comorbid with other diseases, there were 1,036 patients with AD dementia, 664 with CVD/VCI, 778 with LBD (PD/DLB), and 273 with OD, which included 81 patients with TBI, 60 with NPH, 37 with a brain tumor, 26 with FTD, 24 with primary progressive aphasia, and 45 with other diseases. The comparison of demographic characteristics showed significant differences among the different stages of CI (Table 2).

To determine the cutoff scores in different stages, the NC, SCD, or MCI participants without CVD or parkinsonism were included in the AD group. Participants with CVD, VCI without dementia, or VaD were included in the VCI group. Participants with PD, MCI of PD (PDMCI), PDD, or DLB were included in the LBD group. Participants with other brain disorders were included in the OD group.

**Figure 1:**
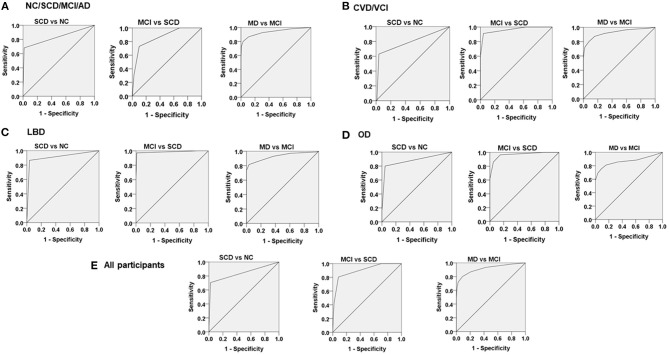
The receiver operating characteristic (ROC) analysis to determine the cutoff scores of the sum of boxes of the Clinical Dementia Rating Scale for normal cognition/subjective cognitive decline (SCD), SCD/mild cognitive impairment (MCI), and MCI/mild dementia among **(A)** the AD group, **(B)** the CVD/VCI group, **(C)** the LBD group, **(D)** the OD group and **(E)** all participants.

The cutoff scores of CDR-SB for NC/SCD, SCD/MCI, and MCI/MD derived from the design phase were applied to the AD group. The ROC analysis showed that the AUC in this group was 0.83, 0.86, and 0.92 for discriminating SCD from NC, MCI from SCD, and MD from MCI, respectively. For the CVD/VCI group, the ROC analysis showed that the AUC in this group was 0.79, 0.96, and 0.93 for discriminating SCD from NC, MCI from SCD, and MD from MCI, respectively. For the LBD group, the ROC analysis showed that the AUC in this group was 0.91, 0.99, and 0.93 for discriminating SCD from NC, MCI from SCD, and MD from MCI, respectively. For the OD group, the ROC analysis showed that the AUC in this group was 0.88, 0.97, and 0.87 for discriminating SCD from NC, MCI from SCD, and MD from MCI, respectively. For all participants, the ROC analysis showed that the AUC in this group was 0.84, 0.90, and 0.92 for discriminating SCD from NC, MCI from SCD, and MD from MCI, respectively (Table 3; Figure 1).

**Table 3:** Comparison of sensitivity, specificity, area under the curve (AUC), and cutoff score among participants with Alzheimer's disease*, vascular cognitive impairment, Lewy body disease, other disorders, and all participants with a sum of boxes of the clinical dementia rating scale in the test phase.

	AD*	**VCI**	**LBD**	**OD**	**All**
*N*	2,666	664	778	273	4,381
SCD vs. NC					
*N*, SCD/NC	544/436	73/77	104/107	20/22	642/741
Sensitivity	0.68	0.63	0.87	0.80	0.71
Specificity	0.98	0.94	0.97	0.95	0.98
AUC	0.83	0.79	0.91	0.88	0.84
AUC 95%CI	0.81–0.86	0.72–0.87	0.87–0.96	0.77–0.99	0.82–0.86
Cutoff	0/0.5	0/0.5	0/0.5	0/0.5	0/0.5
MCI vs. SCD					
*N*, MCI/SCD	650/544	102/73	177/104	57/20	986/741
Sensitivity	0.73	0.91	0.99	0.97	0.81
Specificity	0.90	0.96	0.97	0.85	0.92
AUC	0.86	0.96	0.99	0.97	0.90
AUC 95%CI	0.84–0.89	0.94–0.99	0.97–1.00	0.93–0.99	0.89–0.92
Cutoff	0.5/1.0	0.5/1.0	0.5/1.0	0.5/1.0	0.5/1.0
MD vs. MCI					
*N*, MD/MCI	1036/650	412/102	390/177	174/57	2012/986
Sensitivity	0.74	0.80	0.82	0.81	0.80
Specificity	0.97	0.92	0.97	0.84	0.91
AUC	0.92	0.93	0.93	0.87	0.92
AUC 95%CI	0.91–0.93	0.90–0.95	0.91–0.95	0.82–0.91	0.91–0.93
Cutoff	2.0/2.5	2.5/3.0	2.5/3.0	2.5/3.0	2.0/2.5

AD*, Alzheimer's disease and participants with NC/SCD/MCI without CVD, parkinsonism, or other brain disorders; VCI, vascular cognitive impairment; LBD, Lewy body disease; OD, other disorders; SCD, subjective cognitive decline; NC, normal cognition; MCI, mild cognitive impairment; MD, mild dementia.

Spearman's correlation coefficients among age, education, CDR-SB, MoCA, CASI, NPI, and CI stages are summarized in Table 4. The CDR-SB and CI stages had decreased correlation with age (*r* = 0.43 and 0.38 for CDR-SB and CI, respectively) or education (*r* = −0.34 and −0.31 for CDR-SB and CI, respectively). On the contrary, cognitive tests such as MoCA and CASI both had a moderate correlation with age (*r* = −0.59 and −0.58 for MoCA and CASI, respectively) or education (*r* = 0.62 and 0.54 for MoCA and CASI, respectively), and all had *p* < 0.001. Furthermore, NPI had the highest correlation with CDR-SB (*r* = 0.35, 0.30, −0.15, and −0.15 for CDR-SB, CI stages, MoCA, and CASI, respectively), with *p* < 0.001 (Table 4).

**Table 4:** Spearman's correlation coefficients between age, education, and different diagnostic tools for cognitive impairment.

	**Age**	**Education**	**CDR-SB**	**MoCA**	**CASI**	**NPI**	**CI stages**
Age	1.00						
Education	−0.49^**^	1.00					
CDR-SB	0.43^**^	−0.34^**^	1.00				
MoCA	−0.59^**^	0.62^**^	−0.69^**^	1.00			
CASI	−0.58^**^	0.54^**^	−0.71^**^	0.97^**^	1.00		
NPI	0.04^*^	−0.08^**^	0.35^**^	−0.15^**^	−0.15^**^	1.00	
CI stages	0.38^**^	−0.31^**^	0.83^**^	−0.66^**^	−0.64^**^	0.30^**^	1.00

*CDR-SB, sum of boxes of the Clinical Dementia Rating Scale; MoCA, Montreal Cognitive Assessment; CASI, Cognitive Abilities Screening Instrument; NPI, Neuropsychiatric Inventory; CI stages, cognitive impairment stages grouped using the CDR-SB in all participants*.

* *p < 0.01;*

** *p < 0.001*.

## Discussion

This study has demonstrated several important findings that may provide practical methods for clinical settings and epidemiological research. First, the CDR-SB, NC/SCD, MCI, and dementia were used for the operational diagnosis of the prodromal phase of dementia. In addition, this operational diagnostic method is not only applicable to older adults without comorbidity of a brain or systemic disorder but also suitable for persons with CVD, LBD, or other brain disorders, such as TBI and NPH. Second, all subjects underwent regular neurological examinations, thus ensuring the reliable detection of dementia and cognitive function. Moreover, we used two phases (design and test phases) to validate our hypothesis. The results of the two phases were surprisingly persistent. Furthermore, the data used in this study were sourced from the Show Chwan Dementia registry database, and the sample size was large enough to support the study results. Third, the test group results successfully replicated that of the design group. The findings from both groups were derived from huge study cohorts called the HAICDDS cohorts. These cohorts continue to be followed up, and further longitudinal studies of NC/SC/MCI to dementia progression are warranted and expected. Finally, the universal single-payer healthcare system of Taiwan minimizes barriers to seeking medical treatment (Cheng, 2003; Cheng et al., 2011). This healthcare system decreases selective bias or enrollment difficulty in the study population.

Although AD is the most common cause of dementia, not all types of dementia are AD. Cerebrovascular disease is present in most individuals with dementia, and the dementia risk is increased about 2-fold after stroke (Wolters and Ikram, 2019). Furthermore, VaD is estimated to be ~30% in Asia, somewhat higher than in North America and Europe (Jhoo et al., 2008; Chan et al., 2013). Risk factors for vascular MCI are treatable, and appropriate treatment can prevent or delay dementia progression. Therefore, this group is an excellent candidate for secondary prevention. However, community-dwelling older adults with vascular MCI are often undetected and are not clinically identified until they develop evident dementia (Meguro and Dodge, 2019). LBD, such as PD and DLB, is the second most common degenerative neurological disorder. LBD is a movement disorder and also presents with multi-domain CI throughout the stages of this disease (Emre et al., 2007; Postuma et al., 2015; McKeith et al., 2017). However, CDR and CDR-SB are seldom used in these patients. Other brain disorders, such as NPH, TBI, and FTD, also demonstrated CI in memory, orientation, judgment, and visuospatial functions throughout the stages of these diseases, although the characteristics of these diseases might have some differences. The assessment of most of the cognitive domains is already provided in the CDR Scale; therefore, we propose that the CDR and CDR-SB will help assess the different stages of various brain disorders. This study successfully demonstrates that CDR-SB is practical for detecting dementia and CI with fair accuracy. Moreover, CDR-SB can be reliably applied by non-medical personnel to identify and determine the stage of dementia. This result will be useful for public healthcare and dementia prevention in the community.

Some researchers have proposed that in the context of dementia, the global scale of CDR (CDR-GS) had superior pooled specificity compared with the CDR-SB, while similar sensitivities were demonstrated between the CDR-GS and CDR-SB by Huang et al. (2021). Some crucial factors, such as old age, high educational level, high MCI or dementia prevalence in a developing country, and a lack of observations of informants in these studies, may affect the estimation of the result. In this study, we demonstrated that CDR-SB is sufficient to detect dementia and CI in the pre-dementia stages. Based on the premise of memory, orientation/visuospatial functions, judgment/executive functions, community affairs, home hobbies, and personal care being the most involved not only in AD but also in other subtypes of dementia, we have proposed that the CDR-SB is a good tool and provides an operational diagnostic method. However, we would like to collaborate with other researchers using different languages to validate the findings of this study.

This study had several limitations. First, our study was conducted in only three centers in Taiwan using a hospital-based population. The findings and results of CDR-SB may not be generalizable to all individuals with different stages and disease subtypes. Second, the comparison among the different groups in our study was retrospective and cross-sectional. Therefore, further longitudinal follow-up studies to investigate the dementia progression rates in NC, SCD, and MCI participants are warranted.

## Conclusion

Our findings suggest that the operational determination of SCD, MCI, and dementia using the CDR-SB is practical and can be applied both in clinical settings and on a dementia registration platform. Compared with CASI or MoCA for the CI staging, using CDR-SB was less influenced by age and education.

## Data Availability Statement

The original contributions presented in the study are included in the article/supplementary material, further inquiries can be directed to the corresponding author/s.

## Ethics Statement

The studies involving human participants were reviewed and approved by Show Chwan Memorial Hospital. Written informed consent for participation was not required for this study in accordance with the national legislation and the institutional requirements.

## Author Contributions

Y-WY undertook the literature search and data analysis, edited the author contributions, and was mainly responsible for the revisions and drafts of the manuscript. P-YC participated in the data analysis and contributed to the revisions and final draft of the manuscript. K-CH and C-YW contributed to the revisions of the manuscript. R-CT undertook the literature search and contributed to revisions. All authors contributed to the article and approved the submitted version.

## Funding

This study was financially supported by the Show Chwan Memorial Hospital No. RD-105032 and the research grants from the Ministry of Science and Technology (K-CH, 110-2321-B-039-003- and 110-2314-B-039-010-MY2).

## Conflict of Interest

The authors declare that the research was conducted in the absence of any commercial or financial relationships that could be construed as a potential conflict of interest.

## Publisher's Note

All claims expressed in this article are solely those of the authors and do not necessarily represent those of their affiliated organizations, or those of the publisher, the editors and the reviewers. Any product that may be evaluated in this article, or claim that may be made by its manufacturer, is not guaranteed or endorsed by the publisher.
